# The COVID-19 Pandemic and Associated Depression Among Emergency Medicine Interns: Results from a National Longitudinal Cohort Study

**DOI:** 10.3390/bs15060821

**Published:** 2025-06-15

**Authors:** Carrie Bissell, Lauren Fowler, Destiny Folk, Cortlyn Brown

**Affiliations:** 1 Department of Emergency Medicine, Atrium Health Wake Forest Baptist Hospital, Charlotte, NC 28203, USA; cortlyn.brown@atriumhealth.org; 2 Department of Translational Neuroscience, Wake Forest School of Medicine, Winston-Salem, NC 27101, USA; lafowler@wakehealth.edu; 3 Department of Emergency Medicine, Indiana University School of Medicine, Indianapolis, IN 46202, USA; dfolk@iu.edu

**Keywords:** COVID-19, depression, emergency medicine, residency, medical training, intern

## Abstract

To explore the prevalence of depression among emergency medicine (EM) interns before and during the COVID-19 pandemic. The Intern Health Study is a national longitudinal cohort study examining mental health among interns across various specialties. In this secondary analysis, we focused specifically on EM interns from 2008 to 2022. Participants completed a baseline survey before their intern year and quarterly surveys throughout their intern year. Depression severity was assessed using the Patient Health Questionnaire (PHQ9), with scores of 10 or higher indicating moderate-to-severe depression. In total, 1956 EM interns from 160 programs completed all PHQ9 surveys. PHQ9 scores at baseline (start of the intern year) were significantly lower prior to the COVID-19 pandemic compared to during it (*p* < 0.001). PHQ9 scores at month 9 were significantly higher during the COVID-19 pandemic (*p* < 0.05) compared to pre-COVID-19 pandemic interns at month 9. One-way ANOVA comparing pre-COVID-19 and during COVID-19 differences in PHQ9 from baseline to the end of intern year revealed a significant difference, with during COVID-19 differences being significantly less than pre-COVID-19. There was no significant difference in the proportion of interns with PHQ9 scores greater than 10 during the COVID-19 pandemic. The COVID-19 pandemic had a significant effect on the mental health of EM interns, with higher baseline depression scores observed during the pandemic. However, the smaller change in depression severity over the intern year during the pandemic suggests a complex interplay of factors that warrants further investigation. Our study is the first to examine depression among EM interns during the COVID-19 pandemic using a large, multi-year sample, providing a unique and comprehensive analysis of how the pandemic impacted mental health in this high-risk group. Unlike previous studies with smaller sample sizes, our research offers robust, generalizable insights into the trends and severity of depression in EM interns, highlighting the critical need for ongoing mental health support in medical training.

## 1. Introduction

The prevalence of depression in resident physicians is around 30%, a rate significantly higher than the roughly 10% seen in the general population (Fang et al., 2022). The intern year, the first year of residency training for medical school graduates, is particularly challenging, as many interns have recently moved, left their medical school support system, and encountered the increasing responsibilities and expectations that come with being a physician. Depression during residency, especially if it begins during the intern year, is associated with depression 5 and 10 years later (Kim et al., 2024). Depression also increases the risk of burnout, and is associated with increased motor vehicle accidents and medical errors (de Oliveira et al., 2013). In a previous study, we reported that emergency medicine (EM) interns endorsed increasing levels of depression at month three, as well as at the end of their intern year compared to the start of their intern year (Folk et al., 2024). We also found that higher depression scores were associated with less sleep and increased work hours. In addition, those who were rotating through the intensive care unit (ICU) or who identified as women were also found to report higher levels of depression (Folk et al., 2024).

The literature also suggests a high prevalence of depression amongst healthcare workers during the COVID-19 pandemic, with residents being particularly vulnerable due to high workload and less experience (Pappa et al., 2020; Salari et al., 2020). Only a few small studies exist, however, that examine the effect of the COVID-19 pandemic on depression in EM residents. A study performed in Tehran, Iran, and one in the US found that EM residents reported greater levels of depression during the COVID-19 pandemic (Zangi et al., 2024; Espinosa et al., 2021). To date, no studies, whether large or small-scale, have specifically investigated the impact of the COVID-19 pandemic on depression among emergency medicine interns, despite the distinct challenges encountered during this stage of training and the higher prevalence of depression reported among interns relative to residents across medical specialties. To the best of our knowledge, this study represents the first and only investigation to date, and notably a large-scale one, specifically examining the impact of the COVID-19 pandemic on depression among emergency medicine interns.

Given the essential role of the emergency department in frontline healthcare, understanding interns’ mental health challenges is crucial for ensuring well-being, optimizing patient care, and preventing long-term consequences such as burnout, medical errors, and career dissatisfaction. In the United States, interns play a large role in patient care, decision making, procedures, and documentation, and often function very autonomously. By identifying trends in depression during this period, this study provides valuable data that can inform targeted interventions and institutional policies to better support medical trainees in the face of future healthcare crises.

In this study, we explored two hypotheses. First, we hypothesized that during the COVID-19 pandemic, depression, as characterized by Patient Health Questionnaire (PHQ9) scores, would be higher at each corresponding time point throughout the year compared to pre-pandemic levels. Secondly, we hypothesized that PHQ9 would increase more between the start and end of the year during the pandemic as compared to before the pandemic. 

## 2. Methods

Study Design and Setting: This secondary analysis utilized data from the Intern Health Study, a prospective longitudinal cohort study involving interns from various medical specialties across the United States between 2008 and 2022 (Sen et al., 2010). The University of Michigan managed all recruitment and data collection for the original study. The parent study received approval from the Institutional Review Board at the University of Michigan, Ann Arbor, and was funded by the National Institute of Health (R01MH101459). The parent study adhered to the STROBE guidelines (von Elm et al., 2007). For this secondary analysis, we focused on data from EM interns surveyed during the 2008–2022 period. Interns were excluded from within-subject analyses if they did not complete all surveys during their intern year, but all available surveys were included in the between-subject analyses.

**Figure 1:**
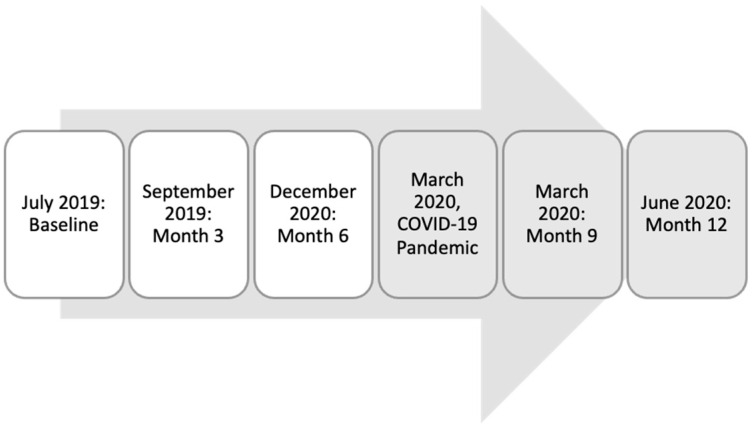
Timeline for 2019 cohort and data considered to be pre- or during the COVID-19 pandemic.

Study Population and Protocol: More than 60 partnering sites across the United States provided contact information for graduating medical students, resulting in 44,221 participants between 2008 and 2022. Additionally, publicly available medical school match lists were used to gather contact information for eligible incoming interns at other institutions. All graduates and incoming interns from these partnering sites between 2008 and 2022 were eligible to participate. Personal email invitations were sent to potential participants. Participants completed a baseline PHQ9 and risk factor analysis using a secure mobile application called MyDataHelps approximately two months before the start of their intern year (baseline) and again at three, six, nine, and twelve months into their intern year. On 11 March 2020, the World Health Organization characterized the COVID-19 outbreak as a pandemic. Thus, for this analysis, we considered PHQ9 scores for the 2019 cohort at baseline, month three, and month six to be pre-COVID-19 pandemic. Months nine and twelve for the 2019 cohorts (March and June 2020) were coded as during the COVID-19 pandemic, as were all subsequent data after June 2020. For the 2019 cohort, PHQ9 scores at baseline, month three, and month six were considered pre-COVID-19, and months nine and twelve were coded as during COVID-19. Longitudinal PHQ-9 assessments of the cohort prior to the COVID-19 pandemic were compared to the cohorts starting in March of 2020. (See Figure 1).

Measures and Outcomes: Demographic information, including age, race, ethnicity, and sex, was collected in surveys given by the parent study. Participants could select from the following options for ethnicity and race: White, Black/African American, Latino/Hispanic, Asian, Native American, Pacific Islander, Other, Multi-racial, and Arab/Middle Eastern. Depression symptoms were categorized based on the PHQ9, which involves nine questions, such as “over the last two weeks, how many days have you had little interest or pleasure in doing things.” Each question is scored from 0 (not at all) to 3 (nearly every day). The sum of all nine questions determines depression severity: none (0–4), mild (5–9), moderate (10–14), moderately severe (15–19), and severe (20 or higher) (Kroenke et al., 2001). Based on a meta-analysis, a PHQ9 score of 10 or greater has a sensitivity of 0.85 (95% I 0.75–091) and a specificity of 0.89 (95% CI 0.83–0.92) for diagnosing major depressive disorder (Manea et al., 2012). For this analysis, we focused on moderate-to-severe depression (PHQ9 scores of 10 or higher), as research indicates that individuals in this category may benefit from a treatment plan that includes counseling and/or therapy (Kroenke et al., 2001).

Data Analysis: Analysis of variance (ANOVA) was used to assess continuous dependent variables (PHQ9) across the independent variable, time. Fisher’s Least Significant Difference post hoc analysis was used for post hoc ANOVA, when warranted. Multivariate analysis of variance (MANOVA) was used to assess multiple dependent variables across time. All statistical significance is reported at *p* < 0.05 or higher. IBM SPSS Statistics Version 29.0 (IBM Corp., Armonk, NY, USA) was used for statistical analysis.

## 3. Results

From 2008 to 2022, 23,929 out of 44,221 (54%) interns across all specialties in the United States participated in the Intern Health Study. Of these, cumulatively, 2203 were EM interns, and of the EM interns, 1123 completed all surveys over the 12-month period. The EM interns represented over 160 different EM programs nationwide.

Among the EM interns who responded to the demographic question (n = 2203), 1216 (55.2%) identified as male and 970 (44%) identified as female (17 participants did not indicate male or female). The ages of the EM interns ranged from 23 to 56, with a mean age of 27.82 (SD = 2.80) and a median age of 27 years. Please refer to Table 1 for detailed participant demographic information.

Self-reported PHQ9 scores were analyzed for participants who completed all surveys, even if individuals did not complete all of the surveys (refer to Table 2). To test hypothesis 1, one-way MANOVA of the PHQ9 scores from before the intern year and every three months found that PHQ9 scores at baseline were significantly lower prior to the COVID-19 pandemic compared to during it: F(1,2038) = 29.258, *p* < 0.001.

**Figure 2:**
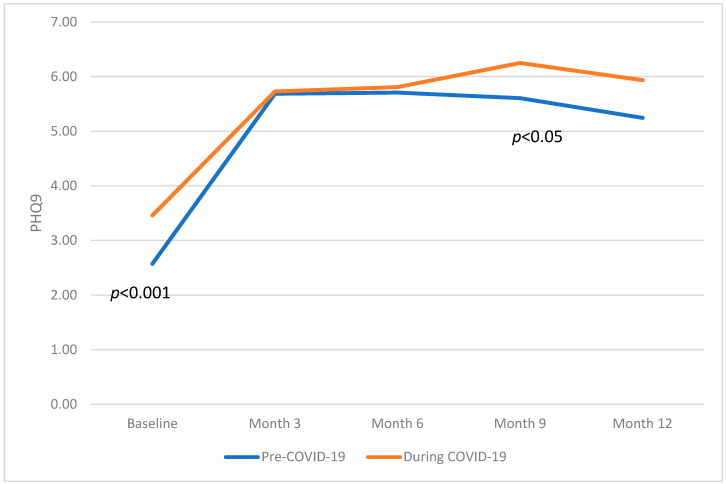
Graphical representation comparing the PHQ9 scores at each time point across the intern year, comparing pre-COVID-19 and during COVID-19.

PHQ9 scores at month nine were significantly higher during COVID-19 (F(1,1288), 4.017, *p* < 0.05) compared to pre-COVID-19. The average PHQ9 scores, confidence intervals, and ANOVA results can be seen in Table 2. A graphical representation comparing the PHQ9 scores at each time point can be found in Figure 2.

PHQ9 scores above 10 were assessed to determine if there were changes in the rates of moderate-to-severe depression pre- vs. during COVID-19. A Chi-Square analysis found no significant difference in the prevalence of PHQ9 scores greater than or equal to 10 pre and during the COVID-19 pandemic at all time points (X^2^ = 2.593, *p* = 0.107). For the proportions of interns with PHQ9 scores of 10 or above at all time points, refer to Table 3, which compares the pre- and during COVID-19 results. The Wilcoxon ranked assessment shows that, at baseline, the instance of PHQ9 scores of 10 or above was significantly lower than at all other time points, for both cohorts (Wilcoxon paired samples ranked assessment (1541) = 12.649, *p* < 0.001).

**Figure 3:**
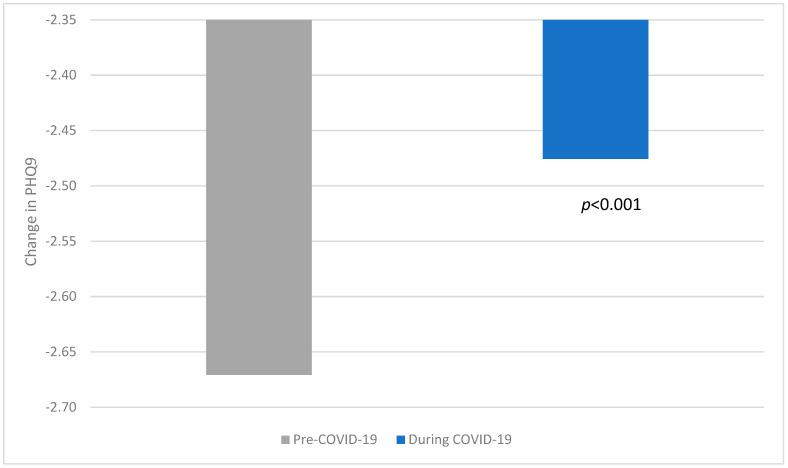
Change in PHQ9 scores across intern year pre-COVID-19 and during COVID-19.

To test hypothesis 2, a one-way ANOVA comparing pre- and during COVID-19 differences in PHQ9 from baseline to the end of the intern year revealed a significant difference in scores, with during COVID-19 changes across the year being significantly less than pre-COVID-19. Thus, the difference between PHQ9 scores at baseline and at month 12 was smaller during COVID-19 than pre-COVID-19 (F (1, 2042) = 10.359, *p* < 0.001). Refer to Figure 3.

In summary, with regard to hypothesis 1, we found that PHQ9 scores at baseline were significantly lower prior to COVID-19 as compared to during the pandemic. We found no significant difference in the prevalence of PHQ9 scores greater than or equal to 10 pre and during the COVID-19 pandemic at any time point. With regard to hypothesis 2, we found that, during COVID-19, PHQ9 changes across the year were significantly less than pre-COVID-19.

## 4. Discussion

In this study, we examined self-reported PHQ9 scores among EM interns from 2008 to 2022, focusing on differences in depression severity before and during the COVID-19 pandemic. Our cohort included 1216 (55.2%) interns who identified as male and 970 (44%) who identified as female (17 participants did not indicate male or female). The ages of the EM interns ranged from 23 to 56, with a mean age of 27.82 (SD = 2.80) and a median age of 27 years. For comparison, the American Academy of Medical Colleges (AAMC) resident reports from 2022 indicate that 61% of all EM residents identified as male and 39% as female (Table B5, 2024, AAMC Data Report). Our primary objective was to explore the prevalence of depression among EM interns before and during the pandemic. We hypothesized that during the COVID-19 pandemic, depression, as characterized by PHQ-9 scores, would be higher at each corresponding time point throughout the year compared to pre-pandemic levels. We found this to be true only at baseline and month 9. Secondly, we hypothesized that PHQ9 would increase more between the start and end of the year during the pandemic as compared to before the pandemic. We found this to be incorrect, with PHQ9 changes during the pandemic being significantly less than before.

First, we found that baseline PHQ9 scores at the start of the year were significantly higher from 2020 (at the start of the pandemic) onwards. This aligns with the existing literature, which has shown that healthcare workers experienced heightened levels of stress and depression during the pandemic due to increased workload, exposure risk, and uncertainties associated with the healthcare crisis (Lai et al., 2020; Pfefferbaum & North, 2020; Shanafelt et al., 2020; Tan et al., 2020). Additionally, our study revealed that PHQ9 scores were significantly higher during the pandemic, further suggesting a sustained impact of the pandemic on depression.

Interestingly, while the baseline PHQ9 scores were higher during the pandemic, the change in PHQ9 scores from the start to the end of the intern year was significantly smaller during COVID-19. This finding contrasts with our hypothesis that depression severity would increase more for interns beginning during COVID-19. One possible explanation for this unexpected result could be the higher baseline depression scores, which may have led to less noticeable changes over time. It is also possible that institutional support mechanisms were enhanced during the pandemic, helping interns manage their depression more effectively despite the challenging circumstances.

Another key finding was that there was no significant difference in the proportion of interns with PHQ9 scores of 10 or above (indicative of moderate-to-severe depression) (Kroenke et al., 2001) before and during the pandemic at any time point. This suggests that while overall depression severity increased, the proportion of interns experiencing moderate-to-severe depression remained stable. This could indicate that the increased baseline scores reflect a general rise in stress and anxiety levels rather than a shift in the severity distribution among those already experiencing significant depression.

To effectively support the mental health of medical trainees in anticipation of future healthcare challenges, institutions should implement comprehensive strategies that encompass both systemic and individual approaches. Establishing robust mental health services, including confidential counseling and peer support programs, can provide trainees with essential resources to manage stress and prevent burnout (Lai et al., 2020; Shanafelt et al., 2020). Integrating wellness curricula that focus on resilience, stress management, and work–life balance into medical education can equip trainees with practical tools to navigate the demands of their profession (Pfefferbaum & North, 2020). Additionally, fostering a culture that prioritizes mental well-being, reduces stigma, and encourages open discussions about depression can create a supportive environment conducive to both personal and professional growth (Tan et al., 2020).

Future research should explore the specific factors contributing to the heightened baseline depression scores during the pandemic and evaluate the effectiveness of different support strategies implemented during this period. Understanding these elements can inform the development of targeted interventions to support the mental health of EM interns and other healthcare professionals during times of crisis.

## 5. Limitations

The study did not record the total number of EM interns invited to participate early on, limiting our ability to calculate specific response rates. More than 60 sites were represented, but we do not know how many interns came from each site. Additionally, the use of self-reported data may introduce bias, and the study design does not allow for causal inferences. Lastly, it is possible that there are confounding factors such as the 2011 ACGME duty hour changes, which limited first-year residents to 16 h shifts with increased supervision and mandatory rest. While intended to reduce fatigue, these changes also led to increased handoffs, reduced autonomy, and disruptions in continuity of care. The 2017 revision, which removed the 16 h cap, may have further impacted stress levels and depression trends independently of COVID-19.

## 6. Conclusions

In conclusion, our study highlights the significant impact of the COVID-19 pandemic on the mental health of EM interns, with higher baseline depression scores observed during the pandemic. However, the smaller change in depression severity over the intern year during the pandemic suggests a complex interplay of factors that warrants further investigation. Continued efforts to monitor and support the mental health of medical trainees are crucial, particularly in the context of ongoing and future healthcare challenges.

## Figures and Tables

**Table 1:** Demographics of emergency medicine intern cohort.

Age	N = 2203	Mean = 27.82 SD = 2.80
Sex		
	Male	1216 (55.19)
	Female	970 (44.030)
	Not indicated	17 (0.77%)
Ethnicity *		
	White	1487 (67.59)
	Black/African American	97 (4.40)
	Hispanic	84 (3.81)
	Asian	293 (13.30)
	Other	235 (10.67)
Participants by Year		
Pre-COVID-19	2008	27 (1.23)
	2009	14 (0.64)
	2010	50 (2.27)
	2011	70 (3.18)
	2012	87 (3.95)
	2013	108 (4.90)
	2014	91 (4.13)
	2015	238 (10.80)
	2016	272 (12.35)
	2017	276 (12.53)
	2018	194 (8.81)
	2019	172 (7.81)
Pre-COVID-19 Total		1599
During COVID-19	2019	85 (3.86)
	2020	219 (9.94)
	2021	121 (5.49)
	2022	90 (4.09)
During COVID-19 Total		515

Data are reported as N(%) unless otherwise specified; 2008–2019 are pre-COVID-19; 2019–2022 are during COVID-19. * Participants could indicate more than one ethnicity, and not all participants indicated ethnicity or sex.

**Table 2:** Average PHQ9 scores for all participants at all time points, pre-COVID-19, and during COVID-19.

	Pre-COVID-19			During COVID-19			Significance
	n	Mean (Stdev)	95% CI	n	Mean (Stdev)	95% CI	
Baseline	1610	2.62 (3.03)	2.47–2.77	430	3.54 (3.43)	3.21–3.88	*p* < 0.001
Month 3	1229	5.71 (5.86)	5.45–5.96	316	5.86 (4.27)	5.38–6.33	*p* > 0.05
Month 6	1110	5.84 (4.59)	5.57–6.11	271	6.01 (4.40)	5.49–6.55	*p* > 0.05
Month 9	960	5.73 (4.59)	5.44–6.02	330	6.33 (4.90)	5.79–6.86	*p* < 0.05
Month 12	893	5.47 (4.56)	5.17–5.77	277	5.94 (4.70)	5.38–6.49	*p* > 0.05

**Table 3:** Percentage of interns with PHQ9 scores of 10 or above at all time points, pre-COVID-19, and during COVID-19.

	Baseline	Month 3	Month 6	Month 9	Month 12
Pre-COVID-19	4.6% (71/1539)	21.4% (217/1012)	24.6% (219/891)	24.7% (202/818)	22.2% (162/731)
During COVID-19	6.7% (27/403)	21.1% (55/261)	30.9% (64/207)	28.9% (81/280)	28.8% (62/215)

## Data Availability

The parent study has an original website available at https://www.internhealthstudy.org/, accessed on 1 May 2023. All data is publicly available at https://www.srijan-sen-lab.com/intern-health-study, accessed on 1 May 2023.
